# Exploring Facilitators and Barriers to STD/STI/HIV Self-Testing Among College Students in the United States: A Scoping Review

**DOI:** 10.1177/21501319241291758

**Published:** 2024-10-22

**Authors:** Jaquetta M. Reeves, Edem Yaw Zigah, Osman W. Shamrock, Dhanyal Khan, Janene Batten, Gamji Rabiu Abu-Ba’are, LaRon E. Nelson, Pascal Djiadeu

**Affiliations:** 1 University of Texas at Arlington, Arlington, TX, USA; 2 Yale University School of Public Health, New Haven, CT, USA; 3 Behavioral, Sexual and Global Health Lab, Jama’a Action, Accra, Ghana; 4 University of Rochester, Rochester, NY, USA; 5 University of Rochester, Rochester, NY, USA; 6 McMaster University, Hamilton, ON, Canada; 7 Yale University Cushing/Whitney Medical Library, New Haven, CT, USA

**Keywords:** STD/STI/HIV testing, self-test kits, college students, U.S., English

## Abstract

**Background:**

HIV affects 1.2 million Americans, with 20% of new diagnoses being 13 to 24-year-olds. Young adult college students are more likely than the general population of 18 to 24-year-olds in the U.S. to engage in sexual practices that increase their risk of STIs.

**Objectives:**

This scoping review explores factors that promote or hinder STD/STI/HIV self-testing among U.S. college students.

**Search Methods:**

A scoping review of original, experimental (randomized or nonrandomized), observational (longitudinal and cross-sectional), and qualitative or mixed-methods U.S. research was conducted using OVID Medline, OVID Embase, PubMed, CINAHL, Web of Science Core Collection, and Cochrane CENTRAL. English-language studies measured STD/STI/HIV self-test kits and college student testing.

**Selection Criteria:**

Inclusion and exclusion criteria were used to narrow down articles that addressed barriers and facilitators to STD/STI/HIV testing, and self-testing among college students in the U.S.

**Results:**

Database searches yielded 8,373 articles. After removing duplicates, 6173 items remained. After independent dual-title/abstract screening, 100 papers were full-text reviewed. Seven retrieved articles were unavailable, and 93 were selected for full-text screening. After reviewing the whole text, 89 papers did not fulfill the inclusion requirements and were deleted, leaving 4 articles in the final analysis.

**Conclusion:**

Additional research on self-testing among college students in the U.S. is urgently required. The results should guide university health policies on the need to cater to the unique requirements of college students by increasing the availability of healthcare and embracing STD/STI/HIV self-testing. This can enhance testing rates, diminish stigmas, and ultimately contribute to wider endeavors to reduce the transmission of infections in the U.S.

## Introduction

In 2021, there were more than 2.5 million reported cases of chlamydia, gonorrhea, and syphilis in the U.S., and the numbers are continuing to increase steadily.^1,2^ The Centers for Disease Control and Prevention (CDC) reported that in the U.S., people aged 15 to 24 years comprise 61% of chlamydia cases and 42% of gonorrhea cases and that the highest numbers of sexually transmitted infections (STIs) are reported among college-aged students.^1,2^ Additionally, adolescents (13-19 years old) and young adults (20-24 years old) make up one-fifth of the estimated 40 000 people diagnosed with human immunodeficiency virus (HIV) annually in the U.S.^3^ The CDC also recently reported that the rate of congenital syphilis has increased tenfold in the last decade.^4^ College-aged individuals, particularly those who have recently gained independence, are more susceptible to infectious diseases such as STIs and HIV infection because they are often in an unsupervised environment with numerous opportunities for a variety of risky behaviors.^4,5^ According to the 2018 National College Health Assessment II, young adult college students have higher rates of sexual behaviors that increase exposure to STIs compared to the general U.S. population of individuals in the 18 to 24 age group.^4,6^ Approximately 1.2 million individuals live with HIV in the U.S., and 20% of new HIV diagnoses are among young people aged 13 to 24 years.^5^ Furthermore, according to the CDC, almost half of young people (13-24) are unaware that they are infected and can pass the virus along with their partner without knowing it.^4^ This issue is further compounded because adolescents and college-aged individuals are less likely to undergo testing because they do not believe they are at risk of contracting an STI.^1,2^ Moreover, college-age students encounter a multitude of barriers regarding access to sexual health services and STD/STI/HIV prevention measures.^2,4,7^ For example, inadequate health insurance coverage, limited employment opportunities, and transportation constraints hinder young people’s ability to seek primary and secondary STD/STI/HIV prevention services.^1,2,6^ Sexually active individuals should undergo annual HIV testing if they engage in high-risk sexual activities during their usual yearly physical examinations.^4,5,8^ However, the avoidance of seeking STD/STI/HIV testing from a primary medical provider is driven by young adults’ concerns regarding privacy, financial implications, stigma, and other negative societal influences.^8-10^ The impact of stigma on STI testing intentions among sexually active college students has been widely studied.^11-13^ To the best of our knowledge, self-testing for STIs/STDs/HIV is one of the methods that can enhance testing rates, identify individuals who are unaware of their infection, and decrease barriers to testing, such as social stigma.^10,14^ Thus, STD/STI/HIV self-testing kits have emerged as an autonomy-supportive solution that offers greater privacy and individual control than clinic-based testing.^15^ Various studies have investigated and demonstrated the acceptability and feasibility of self-testing.^16-19^

Despite the growing availability and acceptability of self-testing kits and their promise to enhance the early detection of STD/STI/HIV cases, a scientific knowledge gap remains regarding the acceptance and utilization of self-testing among young adults, particularly those on college campuses. Although STD/STI/HIV self-testing has previously been examined, the objective of this scoping review was to gain a more comprehensive understanding of the factors that promote or hinder college students in the United States from STD/STI/HIV testing. The research questions directing this review are:

*RQ1*: How does risk perception among college students affect self-testing for STD/STI/HIV?
*RQ2*: What barriers do college students face when considering STD/STI/HIV self-testing?
*RQ3*: What facilitators enhance college students’ willingness to engage in STD/STI/HIV self-testing?

These questions aim to explore the multifaceted factors that influence college students’ decisions to engage in STD/STI/HIV self-testing, focusing on perceptions of risk, barriers, facilitators, and the impact of gender dynamics. The primary objective of this scoping study is to make a valuable contribution to the continuing endeavors of the Ending the HIV Epidemic initiative established by the U.S. Department of Health and Human Services. It also seeks to improve access to sexual health services and enhance strategies for STD/STI/HIV testing among young adults and the broader population.

## Methods

### Search Strategy

**Figure 1.**
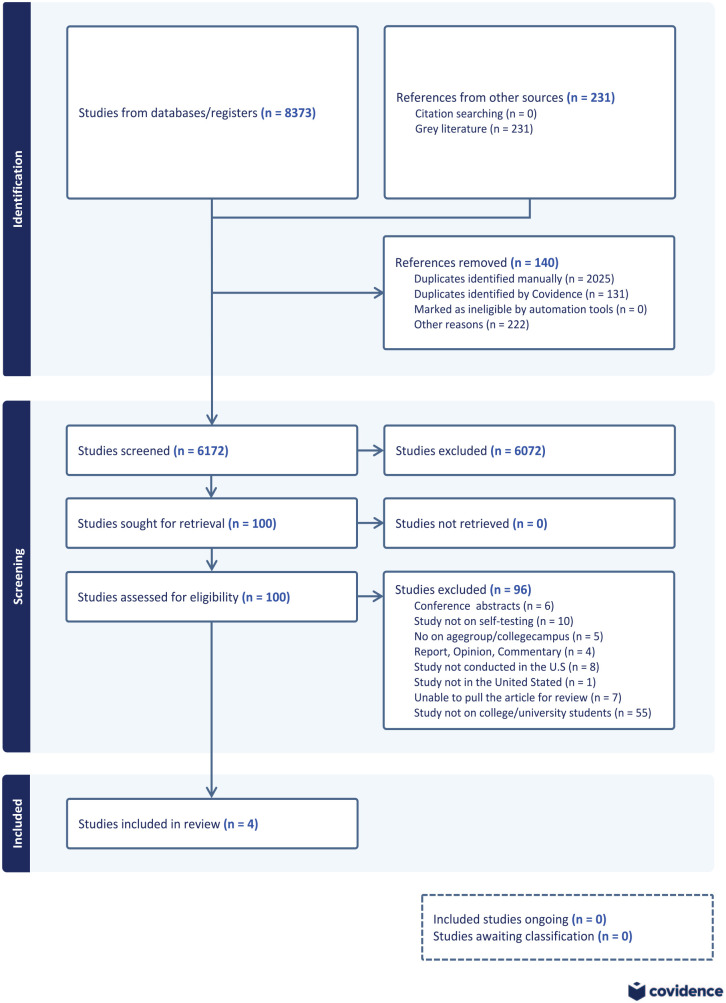
Analysis method for literature selection using PRISM-ScR.

The scoping review was drafted according to the reporting guidance provided in the Preferred Reporting Items for Systematic Reviews and Meta-Analyses Extension for Scoping Reviews (PRISMA-ScR; Figure 1). The PRISMA-ScR checklist^20^ used to prepare this review is presented in Supplemental File 1. The study protocol has been published previously.^21^ Here, we briefly describe these methods. We (JR, DK, EZ, GA-B, LN, OS, PD) used Arksey and O’Malley and later advanced by Levac et al,^22^ Arksey et al.^23^ Reviews were screened in 5 iterative steps to identify the review topic and relevant studies, the studies were selected, the data were charted, and the results were compiled, summarized, and reported.

The search strategy was designed by an expert medical librarian (JB) in consultation with the lead researcher (JR) and then peer-reviewed by a second expert searcher (TM). Following a registered protocol and using the PRISMA-ScR guidelines, the research databases OVID Medline (R) ALL (1946 to July 22, 2024), OVID Embase (1974 to July 22, 2024), PubMed, CINAHL, Web of Science Core Collection, and Cochrane CENTRAL were made. The initial searches were carried out on August 31, 2022, subsequently revised on February 23, 2023, and then reconducted on July 23, 2024. Searches for grey literature were performed in the Policy Commons and ProQuest Dissertations and Thesis databases on March 24, 2023, and subsequently on July 23, 2024. Our analysis encompassed experimental (randomized or nonrandomized), observational (longitudinal and cross-sectional), and qualitative or mixed-methods research conducted in the United States. These studies were published in English and focused on measuring STD/STI/HIV kits and self-testing among college students, without any limitations. The databases were searched using both controlled vocabulary and synonymous free-text words to capture the 3 concepts of HIV or sexually transmitted diseases, self-testing, and college or university students. The search strategies were adjusted for syntax to be appropriate for each database. No limitations, such as language or date range, were applied to the search. The results were uploaded to EndNote (version 20, Thompson Reuters) and deduplicated. The final set was uploaded to Covidence systematic review software (Veritas Health Information) for screening. While the search strategy for 1 database was reported in the published protocol,^21^ the search histories for all databases are provided in Supplemental File 2.

### Inclusion Criteria for This Review

Our study comprised exclusively research carried out in the United States, focusing on undergraduate and graduate students at both 2- and 4-year institutions and universities. Consequently, we exclusively examined English-language publications originating from the United States that assessed STD/STI/HIV kits and self-testing methods among college students.

### Exclusion Criteria for This Review

Our exclusion criteria encompassed review papers (both scope and systematic), book chapters, reports, opinions, commentaries, conference abstracts, and works that were not published in English. Among the studies we examined were experimental (randomized or nonrandomized), observational (longitudinal and cross-sectional), and qualitative or mixed approaches.

### Data Screening

We assigned each article to 2 reviewers to screen eligible studies using Covidence (Veritas Health Innovation, Melbourne, Australia) to screen the titles and abstracts of relevant studies, followed by full-text screening. The reviewers screened 100 abstracts and met the criteria to ensure consistency in the use and clarity of the inclusion and exclusion criteria. Cohen’s kappa statistic was used to measure interrater reliability. Thus, screening was initiated when an agreement of more than 70% was achieved.^24^ Screening was performed independently using JR, LN, DK, EZ, GA-B, OS, and PD. After screening, we conducted data extraction using a Google Form with 2 reviewers assigned to each article and resolved disagreements by consensus in a meeting.

### Data Extraction and Analysis

We extracted data on publication information (eg, authors, year), conceptualizations (eg, questions), methodology, and results. We used Google Forms containing questions for each extractor to ensure a systematic and coordinated data extraction process. Two authors extracted data from each publication, resolved disagreements, and created a single final extraction sheet.

## Results

A total of 8373 items were obtained from the initial search conducted across databases. Following the removal of duplicates, a final count of 6173 articles was preserved. After undergoing separate screening of both titles and abstracts, 100 papers were chosen for a comprehensive assessment of their full texts. Ninety-three articles were selected for full-text screening because 7 retrieved articles were unavailable. After conducting a comprehensive analysis of all the texts, 89 studies were excluded because they did not fulfill the specified criteria for inclusion. Only 4 papers, published in 2018, 2020, 2021, and 2023 were included in the final analysis (Table 1).^10,15,25,26^ The final extraction sheets were collected by 2 writers, who then generated summary tables and completed thematic analysis using Corbin’s grounded theory methods.^27^ The researchers initially individually coded the findings and then systematically examined, merged, and organized the codes into more comprehensive themes for the purpose of writing.

**Table 1.** Literature Review.

Publication title	Authors	Year of publication	Focus/aim	Research method	Data source	Study population/sample size	Findings	Theme
*Creating Innovative Sexually Transmitted Infection* *Testing Options for University Students: The Impact of* *an STI Self-testing Program*	Habel, M.A., Brookmeyer, K.A., Oliver-Veronesi, R., Haffner, M.M.	2018	To analyze CT/GC testing data, assess the acceptability of a self-testing program, and explore trends and positivity.	Observational study with a comparative analysis of clinician- initiated testing and self-testing over two years.	- Laboratory data on CT/GC testing. - Self-administered survey using PDA- based Qualtrics.	Undergraduate college students enrolled in a large university in a rural geographical area. - 2013: 1,014 male and 2,711 female students tested for CT/GC. - 2015: 1,303 male and 3,082 female students tested.	Self-testing significantly increased CT/GC testing positivity among male students from 2013 to 2015, with 31% opting for it. Female students were more likely to test positive than those using clinician testing. Self-testing was particularly popular among male students, and students reported high satisfaction with the program (96.3%).	Facilitator(s): accessibility, convenience, comfort with self-testing, and privacy Barrier(s): perceived safety and low STI risk concerns about accuracy, stigma around STI testing
*Rural College Students’ Amenability Toward Using At-Home Human Immunodeficiency Virus and* *Sexually Transmitted Infection Testing Kits*	Hubach, R.D., Mahaffey, C., Rhoads, K., O’Neill, A.M., Ernst, C., Bui, L.X., Hamrick, J., Giano, Z.	2021	Explore barriers and facilitators to HIV/STI testing among rural college students, the feasibility of at-home testing, and preferred logistical methods for receiving and returning at-home testing kits.	Cross-sectional study.	Online survey questionnaire.	Undergraduate students at a rural state-funded institution - 5,000 invited, 505 accessed survey, 365 completed it, 326 in final analytical sample.	Many students believe they don’t need testing due to being in committed relationships and have low-risk perceptions, lack of knowledge about testing sites, limited free time, insurance coverage concerns, cost barriers, fear of judgment, emotional distress, and confidentiality concerns. They prefer environments that are affirming and convenient, with comfort with a known healthcare provider increasing the likelihood of testing. At-home testing reduces anxiety related to visiting clinics, while in-person testing at a clinic with healthcare provider interaction increases intention. Concerns about test accuracy, potential user error, and clear instructions also influence preferences.	
*Exploring What Influences of Heterosexual College Men and the Practice of Safe Sex: The Power of Stigma and community*	Breny, J.M., Joseph, M., Robledo, D., Rondeau, D., D’Haity, W., Mucha, J., Zapata, P.	2023	To explore the sources of messages that young college men receive regarding safe sex responsibility and sexual decision-making, and how these messages influence their behaviors. The ultimate goal is to inform the development of more effective health promotion programming.	Community- Based Participatory Research (CBPR) with a mixed-methods design.	Surveys and focus groups.	Young adult college men- 6 public health students, 135 male survey respondents, 17 focus group participants.	The study found that 74.1% of participants had sex without a condom, and 66.7% did not consistently use condoms. Non-use was primarily due to alternate birth control, undesirability, and partner preference. 59.3% never tested for STIs, and 60% believed condoms reduced sexual pleasure. The main themes were poor quality condoms, greater concern about pregnancy, lack of awareness, and stigma surrounding STI testing.	
*College students’ comfort with and intention to use self- collection services for STI testing*	Lindley, L.L., Shariff, Chowdhury, T.	2022	Assess college students’ perceptions, comfort, and intention to use self-collection options for STI testing and determine students’ questions or concerns prior to offering these services on campus.	Cross-sectional study.	Online survey via Qualtrics.	Students aged 18 years and older from a large, mid-Atlantic, public university - 434 completed responses out of 504 initiated surveys.	Significant associations between students’ comfort/intention to use self-collection services and their age, perceived STI risk, and previous HIV/STI testing experience. High interest in take-home STI testing kits, particularly among those perceiving low risk.	

### Study Results

The findings revealed that college students’ willingness to engage in self-testing for STDs/STIs/HIV was strongly influenced by their perception of personal risk regarding infection.

Habel et al. conducted a comparative analysis of clinician-initiated testing and self-testing over 2 years (2013-2015) that highlighted the growing trend toward self-testing as a preferred option. In this study, undergraduate students from a large rural university were assessed for their acceptance and uptake of self-testing methods (urine and self-collected vaginal swabs). The research involved a brief self-administered survey conducted among students accessing a university health center between January and December 2015. During this time, 1014 male and 2711 female students were tested for chlamydia and gonorrhea (CT/GC), and in 2015, 1303 male and 3082 female students were tested. Breny et al. carried out a community-based participatory research project to explore heterosexual male college students’ attitudes and behaviors related to safe sex practices. The study aimed to develop effective health promotion messages that encourage safer sexual behavior. Utilizing a mixed-methods design, data were collected via 121 surveys and 5 focus groups held on campus with 17 participants. The focus groups addressed social norms and attitudes regarding STI prevention and condom use. Hubach et al. conducted a cross-sectional study with undergraduate students from a rural, state-funded institution. Participants completed an online survey with open-ended questions to gauge their perceptions and experiences with HIV/STI testing, their willingness to use at-home testing technologies, and their preferences for obtaining at-home testing kits. Of the 5000 students invited to participate, 505 accessed the survey, 365 completed it, and 326 were included in the final analysis. Lindley, Sharif, and Chowdhury’s 2018 study surveyed 434 students from a large public university. The online survey explored students’ sexual behavior, HIV/STI testing practices, perceptions of personal risk, and their comfort and intention to use self-collected STI testing services on campus.

A significant proportion of students expressed concerns about financial barriers, lack of privacy and confidentiality, doubts about the accuracy of self-test results, and potential social stigma. Despite these concerns, self-testing kits were seen as more accessible and easier to obtain, and they provided a more comfortable and less anxiety-inducing testing environment. Gender dynamics also played a critical role, with notable differences between students who chose self-testing and those who did not. Overall, key factors influencing STD/STI/HIV testing among college students in the U.S. can be categorized into 3 primary themes: (1) Perceived personal risk of STD/STI/HIV acquisition, (2) Barriers and facilitators influencing testing decisions, and (3) Students’ experiences and intentions regarding self-testing.

### Perceived Risk of STD/STI/HIV

The perceived risks of STD/STI/HIV among college students influence their decision to undergo self-testing. Studies have indicated that despite being sexually active, most college students perceive themselves as having a low risk of contracting STI or HIV.^15,25,26^ This perception of low risk is a primary factor influencing their decision not to test, as studies have found that college students who never take a self-test often cited their low-risk perception as a reason.^25^ This pattern is particularly true for students involved in multiple monogamous relationships over time.^15^ However, students who identify with the LGBTQ+ community or have multiple sexual partners view themselves as having a moderate- to high-risk perception compared to those with fewer partners, those who identify as heterosexual, or those who have never had an STI.^26^

### Barriers and Facilitators Impacting STD/STI/HIV Testing

One significant finding among college students in STD/STI/HIV testing was the identification of barriers and motivators for self-testing.^15,25,26^ Most students expressed concerns about the various barriers. These included the cost of test kits, the accuracy of test results, confidentiality of test results, and social stigma associated with testing, especially for those living with others who might see the test kit.^10,15,26^ Other identified barriers included user errors in sample collection or test procedures and the potential for sample contamination or loss during return to the health center (after privately taking the sample).^15^ College students who preferred self-testing at a local health facility mentioned 2 additional barriers: the risk of their parents/guardians discovering the test through an insurance bill, compromising confidentiality, and the high transportation costs associated with visiting the health center for the test.^18,26,28^ Despite these barriers, some college students mentioned several facilitators of self-testing. They emphasized the ease of self-testing because it provides a convenient testing option.^25^ Self-testing was also preferred because it offered confidentiality, eliminating the need to set up an appointment at a local health center for sample collection or to know one’s status.^15,25^ The autonomous nature of self-test kits, sample collection, and learning one’s STD/STI/HIV status increases students’ intention to test.^25,26^ Additionally, self-testing provides a more comfortable and less anxiety-inducing testing environment, reducing the stress associated with determining one’s STD/STI/HIV status.^15^

### College Students’ Experiences and Intentions Toward Self-Testing

Several factors influence college students’ intentions to engage in self-testing. These include their age, personal perception of their risk for an STI or HIV infection, and previous experience with self-testing.^26^ College students who have had positive experiences with self-testing over time are more inclined to use self-test kits in the future and are willing to encourage their friends and close relationships to do the same.^25^ Furthermore, an interesting finding emerged when comparing the experiences of college students who chose self-testing versus clinical testing. Students who opted for self-testing were more likely to receive a positive test result than those who chose clinical testing for STD/STI/HIV.^25^ Regarding gender dynamics, there were notable differences between students who opted for self-testing and those who preferred clinical testing. Among female students who chose self-testing, there was a greater likelihood of testing positive for STD/STI/HIV. The primary reason cited for their decision to self-test was unprotected sex.^25^

## Discussion

This scoping review aimed to understand the facilitators and barriers associated with STD/STI/HIV self-testing and uptake among young adults on college campuses in the U.S. We identified 3 eligible studies conducted at public universities. We discovered concerns about how stigma, accessibility, and privacy affect college students’ willingness to self-test for STIs and HIV. College students may under-test for STIs and HIV in large part due to the impression that they are not at high risk for developing these infections.^26^ The prevalence of infections among college students may not be well understood, and they may not prioritize being tested if they think that STIs and HIV are uncommon among their friends or on campus.^25,26^ While some college students believed they were less likely to contract STIs or HIV, our study’s results revealed that students who identified as LGBTQ or who had several sexual partners perceived themselves as being at a greater risk for STI acquisition. Additionally, we found that stigma related to STIs, and HIV prevented college students from being tested or treated for these conditions.^7,10^ Students were deterred from using conventional testing facilities due to fear of being judged, social consequences, repercussions if their parents determined, and misconceptions about these infections.^7^ Globally, self-testing has been shown to reduce the incidence of STD/STI/HIV by increasing testing.^29,30^ Therefore, self-testing can help reduce the impact of stigma by providing a more private and discreet testing option among college students in the U.S. This allows students to maintain confidentiality and avoid judgment by others.^9,26^

Providing convenient and easily accessible STD/STI/HIV testing options increases the likelihood that students will be tested regularly.^4^ Therefore, accessibility is a crucial factor motivating college students to choose self-testing. Despite their usefulness, self-test kits have limitations. However, some students may want additional support, help, guidance, or follow-up care from health professionals. Providing comprehensive sexual health information through campus health services is essential for completing STD/STI/HIV self-testing.

### Strengths and Limitations

This is one of the few scoping reviews that explores factors like facilitators and barriers related to STI and HIV self-testing and uptake among college-aged students in the U.S.

Thorough search parameters and strategies and a variety of databases are among the elements that contribute to the strengths of the present evaluation. All the articles were subjected to comprehensive screening and extraction by several reviewers. The literature was thoroughly examined by reviewing materials from 6 electronic databases and conducting searches for gray literature. Considering the significance of research to surmount the increase in STD/STI/HIV infections among youth and young adults, this study will provide a better understanding of facilitators and barriers to STD/STI/HIV self-testing that could provide insights and directions for research and interventions to increase testing uptake among youth and young adults, thus increasing the early detection of HIV and linkages to care and preventing the spread of HIV. Several limitations were discovered in this investigation. The study findings are predominantly derived from self-reported data obtained from the included studies, which could introduce bias and restrict the reliability of the results. The scarcity of studies can be attributed to the insufficient number of research endeavors specifically targeting college students, despite the abundance of studies on STD/STI/HIV published in the U.S. Therefore, it is difficult to generalize the findings of this scoping review to the general population. However, these findings could aid in understanding the dynamics of STD/STI/HIV testing in the general population and communities worldwide.

## Conclusion

The findings of this scoping review highlight the need for more research focused on STD/STI/HIV self-testing among college students in the U.S. Despite the increasing availability and acceptability of self-testing kits, significant gaps remain in our understanding of their utilization and the factors that influence their adoption within this population. The review highlights the barriers, such as perceived low risk, stigma, privacy concerns, and access issues, that continue to impede the widespread uptake of self-testing. Conversely, the potential facilitators identified—such as autonomy, confidentiality, and convenience—demonstrate the promise of self-testing as a viable strategy for enhancing early detection and prevention efforts. Given the alarming rates of STIs and HIV among young adults, particularly those in college, this review suggests that more targeted research is needed to explore the unique challenges and opportunities for promoting self-testing in this demographic. The findings should inform public health policy and the development of interventions that address the specific needs of college students. By expanding access to and acceptance of self-testing, we can improve testing rates, reduce stigma, and ultimately contribute to the broader efforts to decrease the spread of STIs and HIV in the U.S. and beyond.

## Supplemental Material

sj-pdf-1-jpc-10.1177_21501319241291758 – Supplemental material for Exploring Facilitators and Barriers to STD/STI/HIV Self-Testing Among College Students in the United States: A Scoping Review Supplemental material, sj-pdf-1-jpc-10.1177_21501319241291758 for Exploring Facilitators and Barriers to STD/STI/HIV Self-Testing Among College Students in the United States: A Scoping Review by Jaquetta M. Reeves, Edem Yaw Zigah, Osman W. Shamrock, Dhanyal Khan, Janene Batten, Gamji Rabiu Abu-Ba’are, LaRon E. Nelson and Pascal Djiadeu in Journal of Primary Care & Community Health
